# Effect of Acupuncture on Endometrial Angiogenesis and Uterus Dendritic Cells in COH Rats during Peri-Implantation Period

**DOI:** 10.1155/2017/3647080

**Published:** 2017-05-14

**Authors:** Haoxu Dong, Zhiyan Zhong, Wei Chen, Xiao Wu, Qing Zhang, Guangying Huang, Wei Yang

**Affiliations:** ^1^Institute of Integrated Traditional Chinese and Western Medicine, Tongji Hospital, Tongji Medical College, Huazhong University of Science and Technology, 1095 Jiefang Avenue, Wuhan, Hubei 430030, China; ^2^Reproductive Medicine Center, Tongji Hospital, Tongji Medicine College, Huazhong University of Science and Technology, 1095 Jiefang Avenue, Wuhan, Hubei 430030, China

## Abstract

This study was to explore the mechanism of acupuncture on regulating the endometrial angiogenesis and uterus dendritic cells (uDCs) during the peri-implantation period. Rats, in early pregnancy, were randomized into five groups: normal (N), model (M), acupuncture (A), progesterone (P), and A + P groups. The COH model was established using pregnant mare serum, combined with human chorionic gonadotrophin. Endometrium was collected on days 4, 6, and 8 (D4, D6, and D8) of gestation. Compared with group M, both VEGF and FGF-2 protein and mRNA levels were significantly lower on D4 and higher on D6 and D8 (*P* < 0.05), except for VEGF of group P on D8; the proportion of uterus dendritic cells (uDCs) in the endometrium was significantly lower on D4 and D6 and higher on D8 (*P* < 0.05). In vitro, except for the proliferation of group P on D8, proliferation, tube formation, and migration of uDCs were significantly decreased on D4 and increased on D8 (*P* < 0.05). In addition, acupuncture or progesterone regulated the secretion levels of VEGF, IL-15, and IL-18 secreted by uDCs instead of soluble sFLT-1. In conclusion, acupuncture may regulate angiogenesis of the endometrium and the number and roles of uDCs after COH, and the specific mechanism may be different with progesterone.

## 1. Introduction

During assisted reproductive technologies, controlled ovarian hyperstimulation (COH) is one of the most commonly used methods to induce the development of multiple follicles. However, COH has some disadvantages, including a low embryo implantation rate (20–30% [1]) and a high abortion rate. Studies have shown that, during COH, many factors may result in an imbalance of the internal environment and inhibition of angiogenesis, such as the use of a large number of exogenous gonadotropins, multiple follicular development, and a consistently high level of estrogen [2, 3]. During the peri-implantation period, the process of angiogenesis is considered an important part of the endometrial decidualization. In fact, inhibition of angiogenesis directly affects placentation and may lead to the absorption of embryos, which can result in a reduced pregnancy rate or abortion [4].

During the peri-implantation period, a variety of immune cells, including natural killer cells (NKs), dendritic cells (DCs), and macrophages are involved in the process of angiogenesis [5]. NKs, DCs, and macrophages are involved in the complex and precise regulation of angiogenesis through autocrine or paracrine way. Among them are NKs, which are the most abundant type of lymphocytes in the decidua. And the role of uterine natural killer cell (uNK) in the regulation of angiogenesis has been also the focus of numerous studies [6]. In uNK-deficient mice, abnormalities in placenta development were detected until 10.5 days after gestation [7]. This indicates that uNK may not be central regulators of angiogenesis during decidual implantation. Although the percentage of DCs represents only 1-2% of the total immune cells of the maternal-fetal interface, during pregnancy they play a vital “sentinel” role in both immune activation and tolerance [8, 9]. DCs are potent promoters and core regulators of the adaptive immune response and not only determine whether an effective immune response can occur but also control the type of immune response in the maternal immune system [8]. Therefore, DCs may be critical for angiogenesis during decidual implantation.

Acupuncture has a long history and rich involvement in the treatment of infertility. In recent years, the use of acupuncture during the process of assisted reproduction has increased. Numerous clinical studies have confirmed that the pregnancy rate of patients who received acupuncture treatment during the in vitro fertilization and embryo transfer (IVF-ET) trajectory was significantly increased compared with patients who did not receive acupuncture treatment [10–14]. In traditional Chinese medicine, it is thought that acupuncture causes an opposite regulatory role in the deviation of a normal state and affects the regulation of various immune cells or immune molecules [15]. In our previous study, we have shown that the ratio of mature dendritic cells (CD11c^+^, MHC-II^+^) in COH mice was significantly higher compared to that of mice in a spontaneous pregnancy group (*P* < 0.05) [16]. In addition, in a rat model of implantation failure it was shown that acupuncture could regulate Th1 and Th2 cytokines during the peri-implantation period [17]. Based on the previous findings, we put forward a hypothesis that acupuncture improves endometrial angiogenesis during the peri-implantation period and that the underlying mechanism may involve regulating the number and role of uDCs. The goal of this study was to test the above hypothesis so as to provide a scientific rationale for the application of acupuncture in IVF-ET procedures.

## 2. Materials and Methods

### 2.1. Animals and Grouping

Three hundred SPF grade female virginal Wistar rats (weighing 220–250 g) and 20 SPF grade male adult Wistar rats (weighing 250–300 g) were provided by the Hubei Provincial Center for Disease Control and Prevention, Wuhan, China (the animal certificate SCXK no. 2015-0018). Rats were housed and fed in the animal house barrier system of the Tongji Medical College of Huazhong University of Science and Technology, Wuhan, China (Demonstration of Laboratory Animal Facilities in Hubei Province no. 2010-0057). All animal studies have been approved by the Institutional Animal Care and Use Committee at Tongji Medical College, Huazhong University of Science and Technology, Wuhan, China (IACUC number: 443). After 1 week of adaptive feeding, estrous cycles of female rats were determined by saline vaginal smears. After that, rats were observed for at least 8 days, and those with a normal estrous cycle of 4 days were enrolled in the study. Enrolled rats were randomly divided into the following groups: normal group (N), model group (M), progesterone group (P), acupuncture group (A), and acupuncture plus progesterone group (A + P). Female rats were mated with male rats at 5:00 p.m. with a scale of 1 : 1 or 1 : 2 and checked by the vaginal smear at 8:00 a.m. the next day. When sperm was detected in the vaginal smear, we treated this as the first day (D1) of gestation. Uterine tissues were collected at D4, D6, and D8. In addition, 24 rats were included in each group at D6 and D8. On D4, there were 12 rats. The first five rats of each group on D4, D6 and D8 were used for immunofluorescence (IF), real-time quantitative PCR, Western blot analysis, and flow cytometry (FCM). The remaining rats in each group were used for the purification of uDCs by magnetic bead sorting (MACS).

### 2.2. Reagents and Main Devices

Pregnant mare serum (PMSG) was purchased from the Hangzhou Animal Medicine Factory, China. Human chorionic gonadotrophin (HCG) was provided by Livzon Pharmaceutical Factory, Zhuhai, China. Progesterone injection (Zhejiang Xianju Pharmaceutical Co., Ltd., China) was provided by the pharmacy of Tongji Hospital, Tongji Medical College, Huazhong University of Science and Technology. Vascular endothelial growth factor (VEGF) antibody (sc-7269), fibroblast growth factor 2 (FGF-2) antibody (sc-79), and IL-15 antibody (sc-1296) were purchased from Santa Cruz Biotechnology Company, USA. Anti-dendritic cells antibody [MRC OX-62] (FITC) (ab112196), OX-62 (ab33755), OX-6 (ab23990), goat anti-mouse IgG H&L (ab97035), and donkey anti-rabbit IgG H&L (ab150074) were purchased from Abcam Company, USA. HRP-conjugated rabbit anti-goat IgG (H + L) secondary antibody (GB23204), trypsin digestion (0.25% EDTA, Cat. number G1001), pentobarbital sodium (Cat. number G5003), and crystal violet stain (Cat. number G1014) were purchased from Wuhan Guge Biological Co., Ltd., China. Protease inhibitor cocktail (Cat. number 04693159001) was purchased from ROCHE, Switzerland. HRP-conjugated goat anti-rabbit (Cat. number 074-1506) and HRP-conjugated goat anti-mouse (Cat. number 074-1806) were purchased from KPL Company, USA. RIPA total protein lysis solution (Cat. number AS1004), SDS-PAGE gel preparation kit (Cat. number AS1012), and ECL chemiluminescence detection kit (Cat. number AS1059) were purchased from Wuhan ASPEN Biological Co., Ltd., China. Triton-100 (Cat. number T8200) and Tween-20 (Cat. number T8220) were purchased from Solarbio Technology Co., Ltd., China. DMSO (Cat. number D2650), DAPI (Cat. number D9542), Evans Blue (Cat. number E8010), hyaluronidase (Cat. number H3506), and collagenase IV (Cat. number C5138) were provided by Sigma, USA. Rat lymphocyte separation solution (Cat. number LTS1083) was purchased from Tianjin Hao Yang biological Co., Ltd., China. Thiazolyl blue (CAS: 298-93-1) was purchased from Gen-View Company, USA. RNAiso plus (TaKaRa Code: 9109), RNase-free water (Cat. number 9012), reverse transcription kit (TaKaRa Code: DRR036A), and a SYBR fluorescence quantification kit (TaKaRa Code: DRR420A) were purchased from TaKaRa Biotechnology Co., Ltd., Japan. Modified Roswell Park Memorial Institute (RPMI) Medium 1640 (Cat. number SH30809.01) was purchased from GE Healthcare Life Sciences Hyclone Laboratories, USA. Fetal bovine serum (FBS) was purchased from Gibco (US origin, Ref. number 16000-044, USA). Matrigel basement membrane matrix (Cat number 356234) was purchased from BD Company, USA. Anti-FITC MicroBeads (Cat. number 130-048-701), AutoMACS Running Buffer (Order number 130-091-221), and a MiniMACS Starting Kit (Cat. number 130-090-312) were purchased from Miltenyi Biotec, Germany. Enzyme-linked immunosorbent assay (ELISA) plates, cell strainers (aperture 70 *μ*m, nylon), antibody-coating buffer (Cat. number AR1106-4), VEGF (Cat. number EK0540, Lot. number 2541253523), ELISA kit, and IL-18 ELISA kit (Cat. number EK0592, Lot. number 2971251822) were purchased from Wuhan Boster Biological Engineering Co., Ltd., China. A fms-like tyrosine kinase-1 (Flt-1) ELISA kit was purchased from Raybiotech (Norcross, USA, Cat. number ELR-VEGFR1, Lot. number 1021160776). Recombinant rat IL-15 (Cat. number 400-24) was purchased from BioLegend, USA.

Other materials included filter units (0.22 *μ*m, Merck Millipore Ltd., USA), transwells for 24-well plates (6.5 mm diameter inserts, 8.0 *μ*m pore size, Ref. number 3422, Corning Incorporated, USA), 24- and 96-well cell culture clusters (Corning Incorporated, USA), counting slides (Cat. number 145-0011, Bio-Rad Laboratories, USA), sterile acupuncture needles (Huato, 0.18 × 13 mm, Suzhou Medical Products Factory Co., Ltd., China), Nucleic Acid Protein Analyzer (DU730, Beckman Coulter, USA), Mastercycler gradient PCR apparatus (Eppendorf, Germany), Nikon microimaging system (TE2000-U, Tokyo, Japan), Step-One Real-Time PCR (Applied Biosystems, California, USA), BD FACSCalibur (BD Bioscience, USA), Scanner (LiDE110, Canon, Japan), and microplate reader (BioTek Synergy 2, Vermote, USA). EA.hy926 (human umbilical vein endothelial cells, HUVECs) were derived from the American Type Culture Collection (ATCC) cell bank.

### 2.3. Modeling and Treatment

A total of 1000 IU of PMSG was mixed with 4 ml of special diluent (which was a matching product of PMSG) in a super-clean platform. Similarly, 2000 IU of HCG was mixed with 2 ml of 0.9% sodium chloride. Next, 80 ul of attenuant PMSG (20 IU) was mixed with 0.72 ml of sodium chloride, and 20 ul of attenuant HCG (20 IU) was mixed with 0.78 ml of sodium chloride. Rats in groups M, P, A, and A + P were injected intraperitoneally with PMSG on the day after start of the estrous period (the first day of starting the dioestrus) at 5:00 p.m., then injected with HCG after 48 hours, and immediately mated with male rats. Each rat in group P was injected once a day with 5 mg of progesterone at 4:00 p.m. from D1 to the day of harvesting. Each rat in group A underwent an acupuncture procedure once a day starting from the day of PMSG injection to D4. Rats were restrained in self-made cloth bags and lifted in the air in the process of acupuncture. The acupoints selected were bilateral Zusanli (ST36), Sanyinjiao (SP6), and Taichung (LR3) according the guideline of Hua et al. [18]. The location was as below: ST36 on the back of the knee and 5 mm below the fibula little head, piercing straightly 7 mm; SP6, 10 mm above the inner ankle tip, piercing straightly 4-5 mm; LR3, in the interosseous depression between first and two toes, piercing straightly 1 mm [18]. Sterile acupuncture needles (0.18 × 13 mm) were used. Each acupuncture treatment lasted for 25 minutes, and needles were hand-manipulated every 5 minutes. In groups N, M, and P, rats were fixed in cloth bags from the day of PMSG injection to D4. In group A + P, rats were treated with both progesterone and acupuncture. The rats in group N were injected with a similar dose of saline.

### 2.4. Harvesting

To obtain uterus tissue, rats in each group were anesthetized with 1% pentabarbital sodium by intraperitoneal injection at 4:00 p.m. on D4, D6, and D8. To judge if rats were pregnant, on D6 rats were injected with 0.5 ml 0.5% Evans blue through the tail vein 0.5 h before anesthesia. When present, the blastocysts at D8 were counted and recorded. All uterine tissues of rats on D4, as well as pregnant tissue on D6 and D8, were used for subsequent experiments. Uterine tissues were then split up for multiple analysis. One-sixth uterine tissues of the first five rats of each group were used to prepare frozen sections of 4 *μ*m–8 *μ*m in thickness and stored at −20°C. One-sixth uterine tissues were immediately stored at −80°C and used for Western blot analysis and real-time PCR. The endometrium tissue of the two-thirds of the uterine tissue was scraped out by a bend 20 ml syringe needle and collected to prepare a cell suspension for FCM. The endometrium of the remainder of the uterus samples was prepared as above and collected for preparation of a cell suspension for MACS.

### 2.5. Immunofluorescence Assay

Frozen sections of uterine tissues were taken out from −20°C refrigerator and then placed at room temperature for half an hour to rewarm. Then, sections were fixed in 4% formalin for 10 min and rinsed three times for 5 minutes with PBS. For FGF-2 staining, sections were submerged into a 0.3% Triton X-100 solution for 15 minutes and rinsed three times for 5 minutes with phosphate-buffered saline (PBS). For staining of VEGF and OX-6, this additional step was not performed. Sections were blocked with 5% bovine serum albumin (BSA) (diluted with PBS), incubated with primary antibodies directed against FGF2, VEGF, or OX-6 at 4°C overnight, rinsed with phosphate-buffered saline with Tween (PBST) 5 times for 5 minutes, and incubated with the secondary antibodies (see below) at room temperature for 1 h. The anti-VEGF was used at a 100-fold dilution and OX-6 at a 500-fold dilution, and the goat anti-mouse secondary antibody was diluted 200-fold. In addition, the anti-FGF-2 antibody was diluted in 100-fold, whereas the donkey anti-rabbit secondary antibody was used at a 400-fold dilution. After incubation with the secondary antibodies, sections were rinsed 5 times with PBST (5 minutes each), stained with DAPI (1 : 1500) for 3 minutes, rinsed 3 times with PBST (3 minutes each), and sealed with an anti-fluorescence quenching agent. Images were taken within 24 hours, and sections were stored at 4°C in the dark. Images were analyzed using Image J software.

### 2.6. Western Blot Analysis

Endometrial tissue was obtained as described for FCM under “2.4.* Harvesting,*” homogenized and lysed in tissue protein extraction reagent, supplemented with protease inhibitor cocktail, placed on ice for 30 minutes, and centrifuged at 12,000 rpm for 10 minutes at 4°C. The supernatants were collected and the protein concentration was determined using a BCA protein assay kit. Forty *μ*g of the extracts was mixed with sample buffer, boiled for 10 minutes, and separated on a 12% SDS-page gel for 1.5 h at 100 Volts. Proteins were transferred to a nitrocellulose membrane for 60 minutes at a constant current of 300 mA. Membranes were blocked with 5% nonfat-dry milk for 1 h at room temperature and incubated overnight at 4°C with primary antibodies against *β*-actin, VEGF, or FGF-2 at dilutions of 10,000-fold, 1000-fold, or 500-fold, respectively. Following 5 washes for 5 minutes with PBST, membranes were incubated with HRP-conjugated goat anti-rabbit (10,000-fold dilution for *β*-actin and FGF-2) or HRP-conjugated goat anti-mouse (10,000-fold dilution for VEGF) for 1 h at room temperature. After washing 4 times for 5 minutes with PBST in the dark, membranes were incubated with freshly prepared ECL solution in the dark. Membranes were developed, transferred to films, and analyzed by AlphaEaseFC 4.0, where *β*-actin was used as a loading control.

### 2.7. Real-Time PCR

On D8, embryos were carefully removed using a stereoscopic microscope, and total RNA was extracted from endometrial tissue by Trizol reagent, according to the manufacturer's instructions. RNA purity and concentration were determined by a nucleic Acid/protein analyzer and 2 *μ*g of total RNA was reverse-transcribed using 4 *μ*l of PrimeScript RT Master Mix (5x) in a total reaction volume of 20 *μ*l. Next, the mixture was incubated as follows: 15 minutes at 37°C, 5 seconds at 85°C, and 15 minutes at 4°C. Complementary DNA was frozen and preserved at −80°C prior to PCR amplification. Real-time PCR reactions were performed using an Applied Biosystems Step-One Real-Time PCR System, and the 2^−ΔΔCT^ method was used for data analysis.

  Primer sequences:
   
*β*-actin
      Forward primer: 5′-GACTCATCGTACTCCTGCTTGCTG-3′
      Reverse primer: 5′-GGAGATTACTGCCCTGGCTCCTA-3′
    VEGF
      Forward primer: 5′-GTCCTCACTTGGSTCCCGACA-3′
      Reverse primer: 5′-CCTGGCAGGCAAACAGACTTC-3′
    FGF-2
      Forward primer: 5′-GAGAAGAGCGACCCACACGT-3′
      Reverse primer: 5′-CAGTTCGTTTCAGTGCCACATAC-3′

### 2.8. Flow Cytometry

Endometrium tissue was harvested by scraping out the endometrium as described above. Tissue was collected in 4.8 ml of PBS in disposable aseptic culture dishes and then collected. To digest the tissue, 0.6 ml hyaluronidase (1 mg/ml) and collagenase IV (1 mg/ml) were added and incubated for 2 h in a constant temperature shock box (37°C) [19]. Cells were filtered through a 70 *μ*m cell strainer and centrifuged for 5 minutes at 1500 rpm. The pellet was resuspended in 1 ml of sample diluent and carefully transferred to 10 ml centrifuge tubes which contained 3 ml of lymphocyte separation buffer beforehand. After centrifugation for 30 minutes at 2000 rpm and 18–28°C, cells present in the lymphocyte layer were carefully aspirated, washed with 3 ml washing solution, and then centrifuged for 10 minutes at 2000 rpm. The pellet was resuspended in 90 *μ*l PBS, and 10 *μ*l of diluted OX-62-FITC antibody (10 *μ*g/ml, 1 : 10) was added and incubated in the dark for 30 minutes at 4°C. After washing with 1 ml of PBS and centrifugation for 5 minutes at 1500 rpm, the pellet was resuspended in 100 *μ*l PBS and immediately used for FCM. Data was collected and analyzed with CellQuest and CellQuest Pro software.

### 2.9. Magnetic Beads Isolation of uDCs

**Figure 1:**
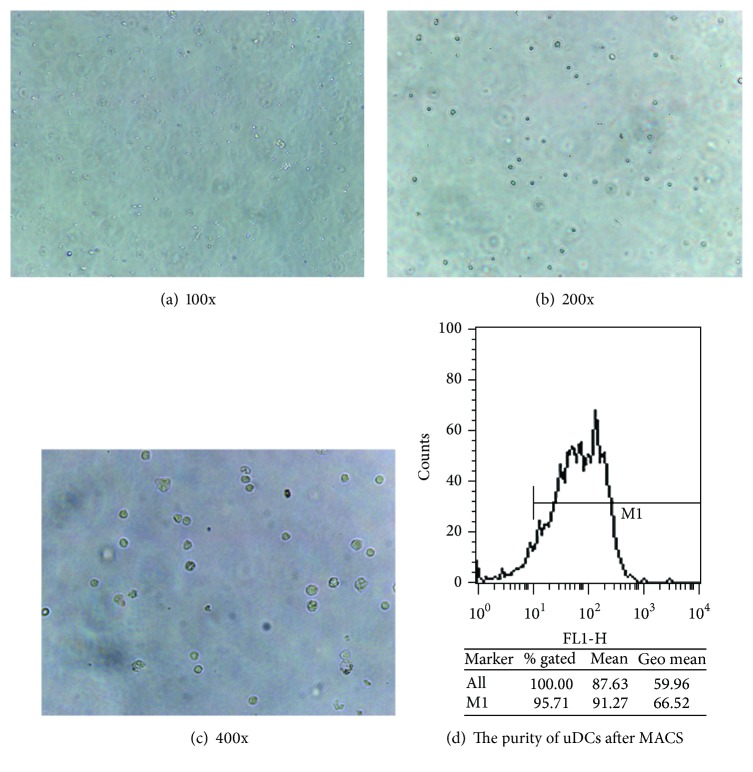
The uDCs and its purity after MACS.

Endometrium tissue was harvested and a single cell suspension was prepared as described above. After incubation with FITC-labeled OX-62 antibody (10 ug/ml, 1 : 10) for 15 minutes at 4°C, cells were washed with 1 ml MACS buffer and centrifuged at 1500 rpm and 4°C for 5 minutes, and the pellet was resuspended in 90 *μ*l MACS buffer and incubated with 10 *μ*l anti-FITC magnetic beads for 15 minutes at 4°C in the dark. The cells were washed, centrifuged, and resuspended in 500 *μ*l MACS buffer. MACS separation was performed as per the manufacturer's instructions. After MACS separation, cells were counted and cultured in 1 ml of complete RPMI medium 1640 (containing 10% FBS) in 24-well plates. Cells harvested from individual rats were cultured separately in different wells. After 48 hours, a migration assay was performed. Cells were trypsinized and cultured in new 24-well plates containing 600 *μ*l of RPMI medium 1640 without serum for another 6 h for the migration assay. Supernatant was collected, filtered through a 0.22 *μ*m filter, aliquoted into 105 *μ*l per tube, and stored at −80°C. In the preexperiment, the purity of uDCs sorted according to the above procedure was over 95% (Figure 1).

#### 2.9.1. MTT Assay

HUVECs were trypsinized with 0.25% trypsin and resuspended in 2 ml of complete RPMI medium 1640 containing 10% FBS. After counting, cells were seeded at a density of 10,000 cells per well in a 96-well plate. Six hours after seeding, the supernatant was removed and 100 *μ*l supernatant derived from uDCs of each group was added in different wells. In addition, five wells received 100 *μ*l of RPMI 1640 medium, without serum, as controls. After incubating at 37°C for 24 hours, 20 *μ*l of MTT reagent was added to each well, and the cells were returned to the 37°C incubator for an additional 4 hours. The supernatant was removed, and 100 *μ*l DMSO was added to each well. The plate, containing the cells, was shaken gently for 10 minutes, after which OD values were measured using a microplate reader at a wavelength of 570 nm. The differences between OD values of experimental versus control wells were used for statistical comparison.

#### 2.9.2. In Vitro Endothelial Migration Assay

Transwell chambers were used to establish a HUVECs and uDCs coculture system. The uDCs were cultured in 24-well plates with 600 *μ*l RPMI 1640 medium (without serum), and 1 × 10^5^ HUVECs were seeded and cultured in the upper transwell chambers containing 100 *μ*l RPMI 1640 medium (without serum) per chamber. Plates were incubated at 37°C for 6 hours under 5% CO_2_. After the incubation, cells on the upper surface of the transwell chambers were carefully removed using swabs, and HUVECs on the lower surface of chambers were fixed by 4% paraformaldehyde for 30 minutes. After 3 washes for 5 minutes with PBS, the lower surface was stained with crystal violet solution for 20 minutes at room temperature, and after a rinse with distilled water, HUVECs were evaluated at a 100x magnification. From each chamber, pictures were taken from 10 random fields. The average number of migrating cells was calculated using Image-Pro Plus 7.0 software.

#### 2.9.3. Umbilical Vein Endothelial Tube Formation

Prior to the tube formation test, all materials which would have contact with Matrigel was precooled at −20°C. Matrigel was melted at 4°C, and 50 *μ*l of Matrigel was added per well of a 96-well plate. The plate was then incubated for 1 hour at 37°C to allow gelatinization to occur. HUVECs (3.5 × 10^4^ per well) were resuspended in uDCs supernatant and transferred to the wells containing Matrigel. The plates were incubated at 37°C for 24 hours and images were taken at a magnification of 40x. Images were analyzed using Image-Pro Plus 7.0 software.

#### 2.9.4. Enzyme-Linked Immunosorbent Assay

Levels of VEGF, soluble fms-like tyrosine kinase-1 (sFLT-1), IL-15, and IL-18 of uDCs were analyzed by ELISA. The sensitivity of the VEGF and IL-18 ELISA kits was <1.0 pg/ml, and none of the samples had a cytokine level of >1000 or <16.9 pg/ml. There was no cross-reactivity of VEGF and IL-18 with other cytokines. The sensitivity of Flt-1 ELISA kits was 0.41 ng/ml, the interassay coefficients of variation (CV%) were <12%, and the intra-assay CV% was <10%. None of the samples had a cytokine level of >100 or <0.41 pg/ml.

The IL-15 was performed as follows: the primary antibody directed against IL-15 was diluted 2000-fold with antibody-coating solution, and 100 *μ*l was added to each well of an ELISA plate. The plate was sealed with a sealing membrane and stored overnight at 4°C. Next, the plate was washed 3 times with 300 *μ*l PBST (5 minutes each), blocked with 200 *μ*l 5% BSA for 2 hours at 37°C, washed 3 times with 300 *μ*l PBST (5 minutes each), and added to 100 *μ*l of the uDC supernatant. Plates were incubated at 37°C for 1.5 hours and washed as described above, and 100 *μ*l of HRP-conjugated rabbit anti-goat (1 : 2000) was added and incubated at 37°C for 30 minutes. Plates were washed as above and 100 *μ*l of TMB solution was added and incubated at 37°C for 30 minutes. Next, 100 *μ*l of TMB stop solution was added and the optical density of each well was determined by a microplate reader at 450 nm. Different concentrations of recombinant rat IL-15 were included as a standard control.

### 2.10. Statistical Analysis

All data was presented as mean ± SD or percentage (%). Data was analyzed by SPSS 22.0 software. Results were analyzed using the one-way factorial analysis of variance (ANOVA), followed by Dunnett's T3 test for data with equal variances not assumed or by the Chi-square test. *P* < 0.05 was considered statistically significant.

## 3. Results

### 3.1. Mating Rate, Pregnancy Rate, and Embryo Number

**Figure 2:**
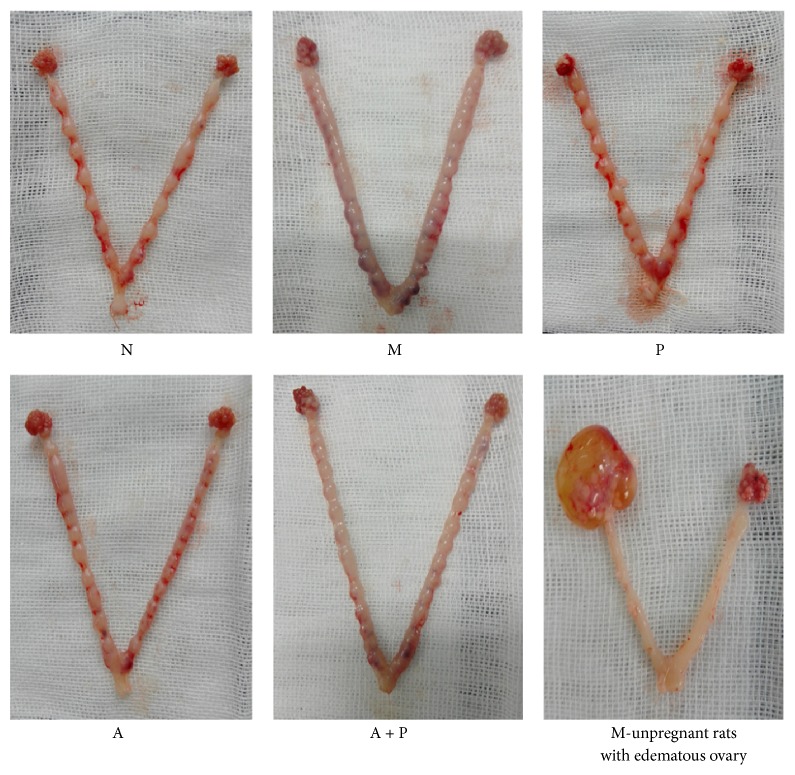
The uterus and ovaries of rats at the eighth day after fertilization.

We did not find a statistical significant difference in the mating rate between groups (*P* > 0.05). Rats on D6 and D8 were used to calculate the pregnancy rate. Pregnancy rate of group M was significantly lower when compared to group N (*P* < 0.01). In addition, compared with group M, the pregnancy rate of groups P, A, and A + P was significantly higher (*P* < 0.05 and *P* < 0.01, resp.). Given that the Evans blue method could not accurately determine the number of embryos, only D8 rats were used to calculate the embryo number. Moreover, compared with group M, the embryo number of groups N, P, A, and A + P was significantly lower (*P* < 0.01). In two cases (one in group M, the other in group P) edematous ovaries were found (Figure 2 and Table 1).

### 3.2. Distribution of OX-6 in the Endometrium

**Figure 3:**
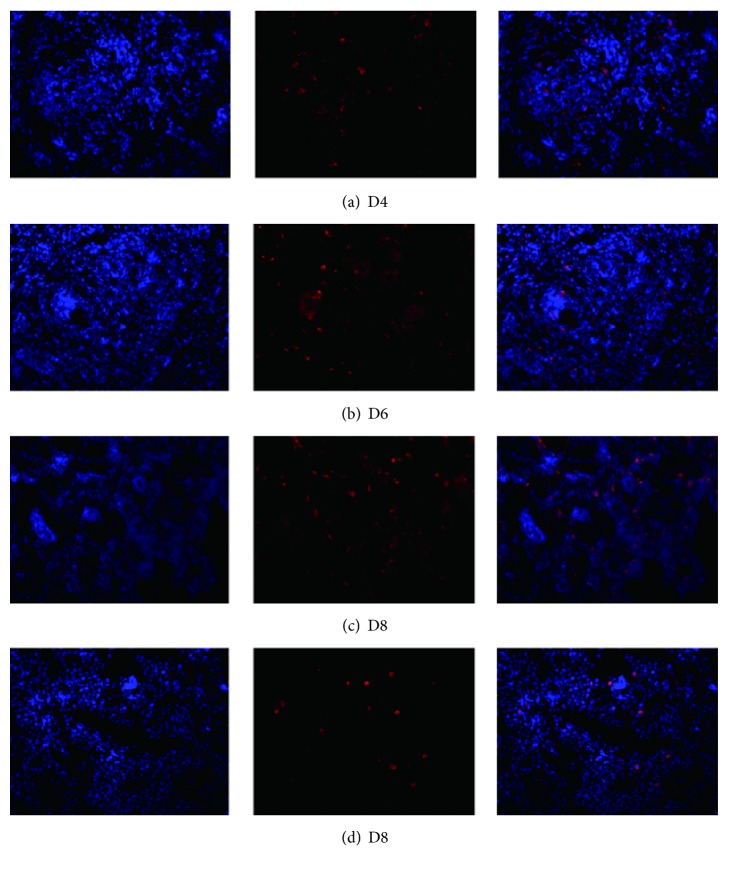
Distribution of OX-6 in the endometrium: (a) D4, the vicinity blood vessels; (b) D6, the vicinity blood vessels; (c) D8, the maternal-fetal interface; (d) D8, the embryo. Original magnification: ×200.

Because we failed to localize uDCs using OX-62, we used OX-6, an anti-MHC Class II, as an alternative way of locating uDCs. OX-6 can recognize a monomorphic determinant of dendritic cells, B lymphocytes, and a variety of macrophages. We found that, on D4 and D6, OX-6 positive cells accumulated near blood vessels, whereas on D8 they were found near the maternal-fetal interface and in embryos (Figure 3).

### 3.3. Expression of Endometrial VEGF and FGF-2 Protein

#### 3.3.1. Immunofluorescence

**Figure 4:**
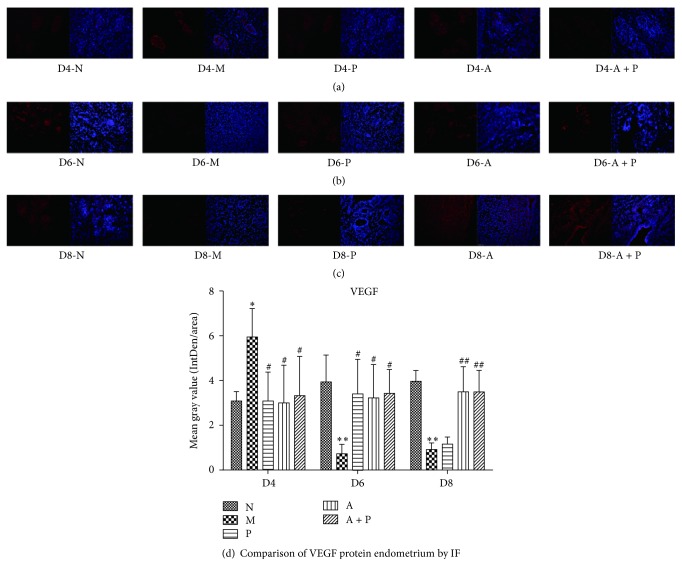
Expression of endometrial VEGF protein: (a) D4; (b) D6; (c) D8; and (d) comparison of VEGF protein by mean gray value (*n* = 3). *∗* or *∗∗* represents that there is significant difference between M and N groups (*P* < 0.05 or *P* < 0.01). # or ## represents that there is significant difference compared with M group (*P* < 0.05 or *P* < 0.01). Original magnification ×200.

**Figure 5:**
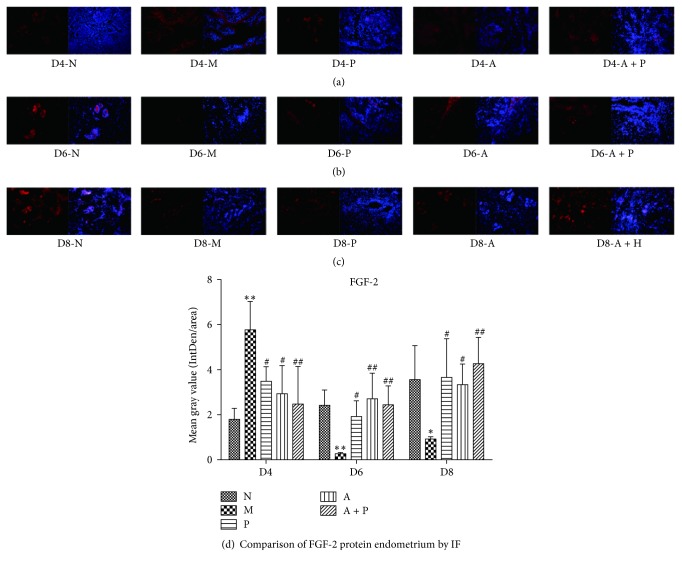
Expression of endometrial FGF-2 protein: (a) D4; (b) D6; (c) D8; and (d) comparison of FGF-2 protein by mean gray value (*n* = 3). *∗* or  *∗∗* represents that there is significant difference between M and N groups (*P* < 0.05 or *P* < 0.01). # or ## represents that there is significant difference compared with M group (*P* < 0.05 or *P* < 0.01). Original magnification ×200.

When compared to group N, the expression of endometrial VEGF and FGF-2 in group M was significantly higher on D4 (*P* < 0.05 and *P* < 0.01, resp.), whereas on D6 and D8, the expression was significantly lower (*P* < 0.05 and *P* < 0.01, resp.). In addition, compared with group M, endometrial VEGF and FGF-2 protein in groups P, A, and A + P was significantly lower on D4 (*P* < 0.05 and *P* < 0.01, resp.) and higher on D6 and D8 (*P* < 0.05 and *P* < 0.01 resp.), except for VEGF in group P on D8 (*P* > 0.05, Figures 4 and 5).

#### 3.3.2. Western Blot Analysis

**Figure 6:**
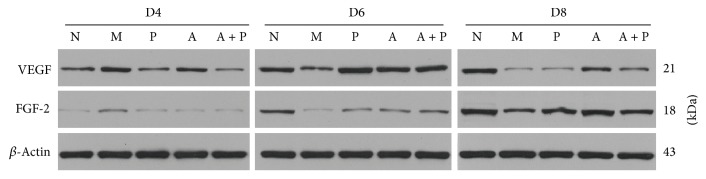
Expression of endometrial VEGF and FGF-2 proteins on D4, D6, and D8 by Western blot.

Compared with group M, the expression of endometrial VEGF and FGF-2 protein in groups N, P, A, and A + P was significantly lower on D4 (*P* < 0.05) and higher on D6 and D8 (*P* < 0.05), except for VEGF in group P on D8, which was not significantly different (*P* > 0.05, Figure 6).

### 3.4. Expression of Endometrial VEGF and FGF-2 mRNA

**Figure 7:**
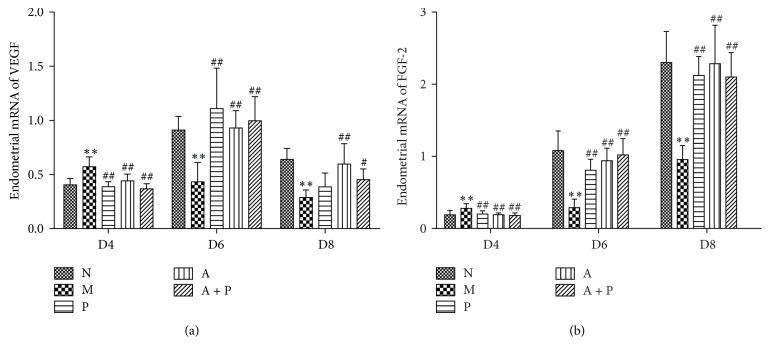
Expression of endometrial mRNA: (a) VEGF (*n* = 5) and (b) FGF-2 (*n* = 5). *∗∗* represents that there is significant difference between M and N groups (*P* < 0.01). # or ## represents that there is significant difference compared with M group (*P* < 0.05 or *P* < 0.01).

The expression of endometrial VEGF and FGF-2 mRNA in groups N, P, A, and A + P was significantly lower on D4, when compared to group M (*P* < 0.01). In addition, on D6 and D8 the expression was higher (*P* < 0.01 and *P* < 0.05, resp.), except for VEGF mRNA in group P on D8, which was not significantly different (*P* > 0.05, Figure 7).

### 3.5. Expression Rate of uDCs by Flow Cytometry

**Figure 8:**
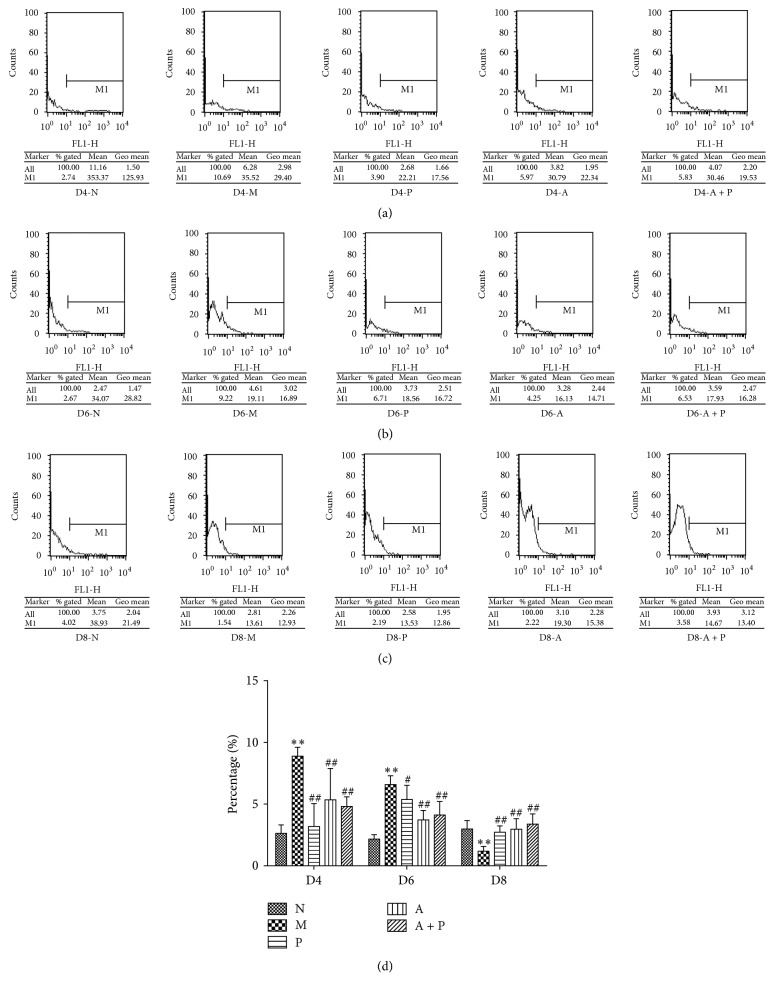
The proportion of uDCs in lymphocytes of the endometrium of pregnant rats (*n* = 5). *∗∗* represents that there is significant difference between M and N groups (*P* < 0.01). # or ## represents that there is significant difference compared with M group (*P* < 0.05 or *P* < 0.01).

Compared with group M, the expression rate of uDCs in groups N, P, A, and A + P was significantly lower on D4 and D6 (*P* < 0.01 and *P* < 0.05, resp.) and higher on D8 (*P* < 0.01, Figure 8 and Table 2).

### 3.6. Lymphocytes Content Before and After MACS

No significant differences were observed in the number of lymphocytes prior to MACS separation among the five groups on D4, D6, and D8 (Table 3). The number of uDCs after MACS separation was below the detection limit of the cell counter used (1 × 10^5^ cells).

### 3.7. Proliferation of Human Umbilical Vein Cells

**Figure 9:**
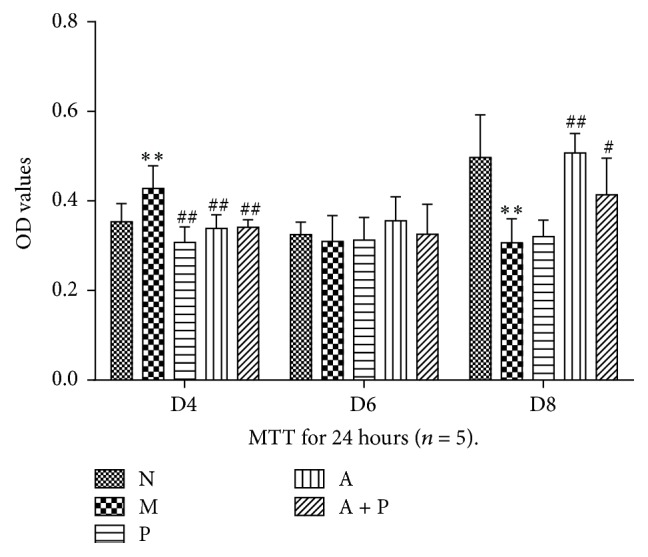
The proliferation of HUVECs culturing by uDCs culture supernatant. *∗∗* represents that there is significant difference between M and N groups (*P* < 0.01). # or ## represents that there is significant difference compared with M group (*P* < 0.05 or *P* < 0.01).

Compared with group M, the proliferation of groups N, P, A, and A + P on D4 was significantly lower (*P* < 0.01). On D6, no significant differences were observed among the five groups (*P* > 0.05). However, compared with group M, the proliferation of groups N, A, and A + P on D8 was significantly higher (*P* < 0.01 or *P* < 0.05). Moreover, on D8, no significant differences were observed between groups P and M (*P* > 0.05, Figure 9).

### 3.8. In Vitro Endothelial Migration Assay

**Figure 10:**
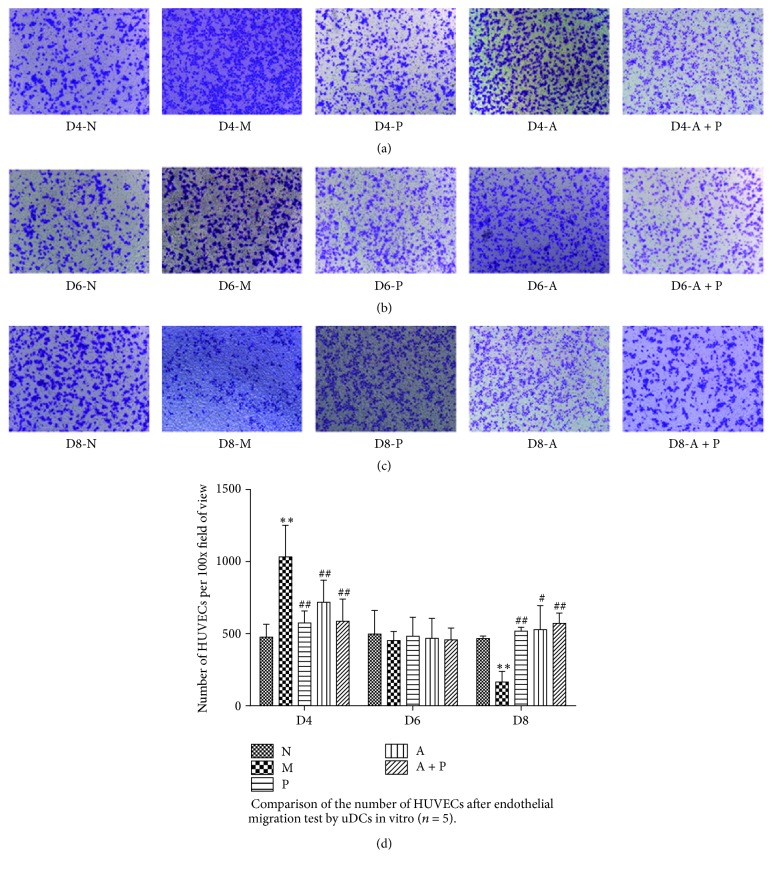
Comparison of the number of HUVECs after endothelial migration by uDCs in vitro. *∗∗* represents that there is significant difference between M and N groups (*P* < 0.01). # or ## represents that there is significant difference compared with M group (*P* < 0.05 or *P* < 0.01).

Compared with group M, the average number of HUVECs per field of view of groups N, P, A, and A + P on D4 was significantly lower (*P* < 0.01). In addition, no significant differences were found among the five groups on D6 (*P* > 0.05). Compared with group M, the average cell number of groups N, P, A, and A + P was significantly higher (*P* < 0.01 and *P* < 0.05, resp., Figure 10 and Table 4).

### 3.9. Umbilical Vein Endothelial Tube Formation

**Figure 11:**
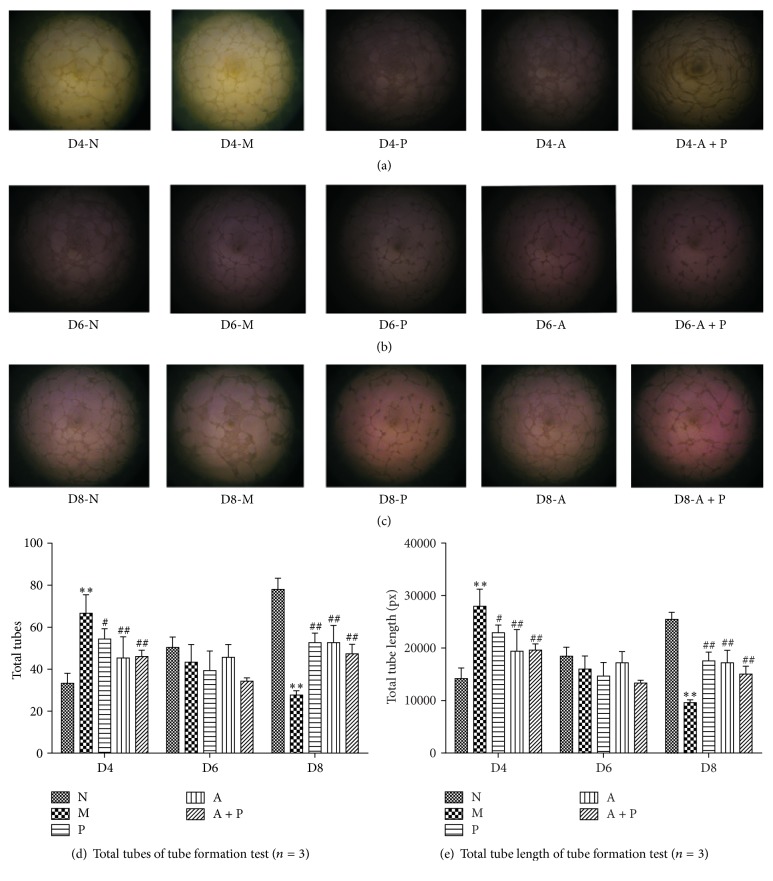
Umbilical vein endothelial tube formation test of uDCs culture supernatant. (a) Selected picture in D4 groups; (b) selected picture in the D6 groups; (c) selected picture in D8; (d) total tubes; (e) total tubes length. ((d) and (e)) *∗∗* represents that there is significant difference between M and N groups (*P* < 0.01). # or ## represents that there is significant difference compared with M group (*P* < 0.05 or *P* < 0.01).

Compared with group M, the total number of tubes and the total tube length of the cells in groups N, P, A, and A + P on D4 were significantly lower (*P* < 0.01 and *P* < 0.05, resp.). No significant differences were found among the five groups on D6 (*P* > 0.05). In addition, compared with group M, the total number of tubes and the total tube length of group N, P, A, and A + P on D8 were significantly higher (*P* < 0.01, Figure 11 and Table 5).

### 3.10. Expression of VEGF, sFlt-1, IL-15, and IL-18 In Vitro

Compared with group M, the secretion levels of VEGF in groups N, P, A, and A + P on D4 were significantly lower (*P* < 0.01). On D6, no significant difference was observed among the five groups (*P* > 0.05). However, compared with group M on D8, the levels of VEGF in groups N, P, A, and A + P were significantly higher (*P* < 0.01 and *P* < 0.05, resp.). Moreover, no significant differences in sFlt-1 levels were observed among the five groups on D4, D6, and D8 (*P* > 0.05).

**Figure 12:**
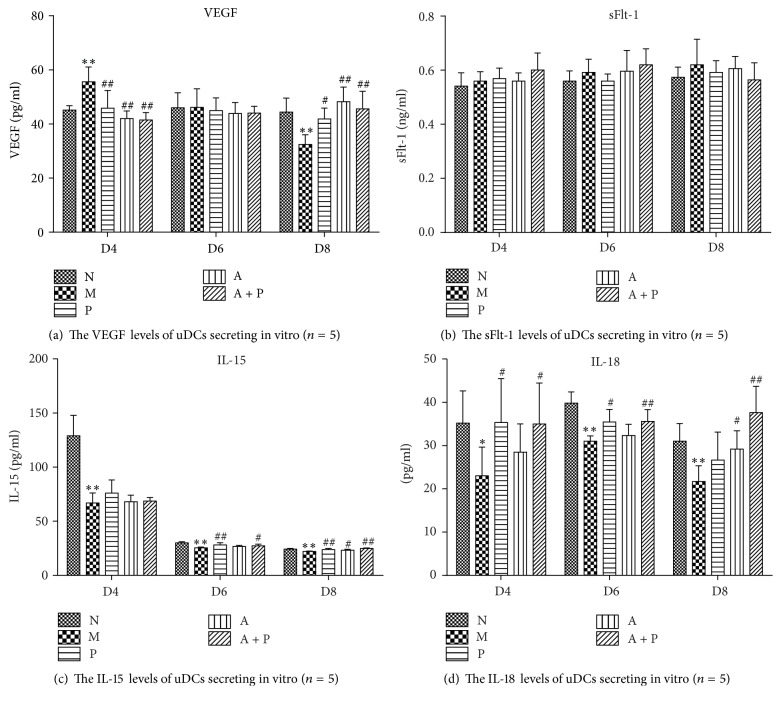
Expression of the levels of (a) VEGF, (b) sFlt-1, (c) IL-15, and (d) IL-18 uDCs secreting in vitro. *∗* or *∗∗* represents that there is significant difference between M and N groups (*P* < 0.05 or *P* < 0.01). # or ## represents that there is significant difference compared with M group (*P* < 0.05 or *P* < 0.01).

Compared with group N, the secretion levels of IL-15 in group M on D4, D6, and D8 were significantly lower (*P* < 0.01). On D4, no significant differences were found in groups P, A, or A + P when compared with group M (*P* > 0.05). Compared with group M, the levels of IL-15 of groups N, P, and A + P on D6 and groups N, P, A, and A + P on D8 were significantly higher (*P* < 0.05 and *P* < 0.01, resp.). There was no significant difference between groups A and M on D6 (*P* > 0.05). In addition, compared with group N, IL-18 levels in group M on D4, D6, and D8 were significantly lower (*P* < 0.05 and *P* < 0.01, resp.). Compared with group M, IL-18 levels in groups P, and A + P on D4 and D6, and in groups A and A + P on D8 were significantly higher (*P* < 0.05 and *P* < 0.01, resp.). No significant differences were found in group A on D4 and D6 and in group P on D8, when compared with group M (*P* > 0.05, Figure 12 and Table 6).

## 4. Discussion

The results of the pregnancy rate as found in our study are consistent with those published in clinical studies [10–14]. After COH, the number of implanted embryos was significantly higher, whereas the pregnancy rate was significantly lower. After treatment with acupuncture or progesterone, the number of implanted embryos decreased, and the pregnancy rate increased. Progesterone is often used in the clinic to provide luteum support in patients with COH and to reduce the risk of luteal phase deficiency (LPD) [20]. Given that acupuncture during IVF is often used in combination with progesterone, we designed the A + P group to verify the therapeutic effect of acupuncture. Our acupuncture approach ranges from the start of PMSG to D5, which is consistent with clinical treatment. In addition, the side effects of acupuncture treatment after embryo implantation were avoided. Although the effect of acupuncture contributing to the success of IVF in humans remains debatable [21], the results in our rat study are promising. The reproductive ability in rats is very high, and the pregnancy rate in the N group was 100%. Furthermore, rats with double uteruses have a relatively large uterine area (the highest embryonic number of unilateral uteruses in group M was 36, which is 3 times that of the mean number of embryos in normal rats). In addition, the sample size (*n* = 226) that was used to calculate the pregnancy rate in rats was sufficient. Therefore, we believe that the effect seen in our A, P, and A + P rats is real.

Our VEGF and FGF-2 studies verified the hypothesis that acupuncture can improve endometrial angiogenesis in COH rats during the peri-implantation period of pregnancy. During angiogenesis, endothelial cells proliferate from existing blood vessels and then migrate and differentiate to form new capillaries. Under stable conditions, the balance between angiogenic and antiangiogenic factors is maintained. However, under conditions, such as decidualization and placentation, positive regulators of angiogenesis play a leading role in the activation of endothelial cells, which promotes vascularization. Different than decidualization, which is induced by nonspecific stimulation, decidual vascular remodeling and growth only occur during pregnancy and are regulated by a variety of factors from both maternal and embryonic matter. Among these, VEGF and FGF-2 are important growth factors that promote the process of angiogenesis. VEGF plays a role in almost all angiogenic processes during the peri-implantation period, including vasodilatation, endothelial cell proliferation, migration, and tube formation [22] through the autocrine or paracrine system, and FGF-2 is a potent mitogen for endothelial cells [23]. Previous studies have shown that endometrial VEGF of the COH group on D3, D4, and D5 was increased compared to that in normal rats [24]. Our data also demonstrated that on D4, the mRNA and protein levels of VEGF and FGF-2 in COH rats were significantly higher compared to that of group N. However, on D6 and D8 the expression of VEGF and FGF-2 was significantly lower. Interestingly, during the peri-implantation period FGF-2 mRNA expression was slightly increased, which was not the case for VEGF. We found that the VEGF mRNA expression in group N during the peri-implantation period first increased and then decreased. That is, the peak of VEGF mRNA expression in group N was found on D6, whereas the peak for VEGF mRNA levels in group M occurred on D4. We hypothesize that the endometrial receptivity was significantly reduced after COH [25] and that pinopodes emerge prematurely, leading to early opening of the implant window [26]. Subsequently, the peak in angiogenesis, which should appear on D5 when embryos implant, may be in advance. A number of researchers put forward the concept of the estradiol (E_2_)/P ratio and believe that the appropriate ratio determines the ratio of glycosyl conjugates on the surface of the endometrial epithelial, which during the COH cycle causes the high E_2_/P ratio to lead to an imbalance in glycosyl conjugates, which results in a decrease of endometrial receptivity [27, 28]. Our results also demonstrated that the supplementation of progesterone on COH rats benefits embryo implantation by regulating mRNA and protein expression of VEGF and FGF-2 during the peri-implantation period. However, progesterone treatment did not improve the expression of VEGF on D8. In contrast, acupuncture did regulate the mRNA and protein expression of both VEGF and FGF-2, suggesting that the underlying mechanisms of progesterone and acupuncture are different. Although not supported by animal studies, electroacupuncture could decrease the serum VEGF levels of patients on the day of ovum pick-up, hCG, and embryo transfer [29]. Our results indicate that acupuncture decreases the exorbitant VEGF and FGF-2 levels of endometrium in COH rats on D4, while it increases the relatively low levels on D6 and D8.

Our FCM data indicates that acupuncture regulates the number of uDCs. VEGF and FGF-2 are important growth factors that are involved in regulating the angiogenic process at the fetal-maternal interface during peri-implantation [23]. During the peri-implantation period, mouse uDCs were located near blood vessels, where they produce VEGF [30]. DCs and decidual DCs express MHC-II in all stages of pregnancy. In our study, we found that, on D4 and D6, OX-6 positive cells were also detected near blood vessels. In humans, most decidual DCs, in early pregnancy, are immature DCs (DC-SIGN^+^ cells), and a small proportion of DC-SIGN^+^ cells are associated with spontaneous abortion [31]. On the contrary, a high proportion of mature DCs is often associated with higher abortion rates [32]. Both immature and mature DCs could be identified by targeting OX-62, which is a relatively specific antigen that is expressed on DCs in rats [33]. Based on our FCM data, the expression of uDCs in group M was significantly increased on D4 and D6 when compared to group N, whereas on D8, the expression was abnormally decreased. The variation between D4 and D8 was also seen for expression of VEGF and FGF-2, which indicated that, during peri-implantation, uDCs are important players in the process of angiogenesis. In contrast, the expression of uDC in group M on D6 was abnormally high and did not synchronize with expression of VEGF and FGF-2. We speculate that this difference may be due to increased expression of mature DCs on D6. In addition, our results indicate that both acupuncture and progesterone are involved in regulating the expression of uDCs.

In vitro, we demonstrated the ability of HUVECs to proliferate, form formations, and migrate when cocultured with uDCs. To evaluate if acupuncture regulates the function of uDCs, we evaluated levels of VEGF, sFLt-1, IL-15, and IL-18 that were secreted by uDCs. We found that the data on proliferation, tube formation, and migration were consistent, and there were no significant differences among the five groups on D6. In group M, proliferation, tube formation, and migration were significantly increased on D4, whereas on D8, this was decreased. Both acupuncture and progesterone regulated proliferation, tube formation, and migration of uDCs. Prior to MACS, no significant differences were observed in the number of lymphocytes among the five groups on D4, D6, and D8; therefore, based on the FCM data, we evaluated the number of uDCs after MACS. Variation in the number of uDCs on D4 and D8 was similar for migration, tube formation, and migration test and different on D6. This indicated that perhaps the number of uDCs played a major role in migration, tube formation, and migration of HUVECs on D4 and D8. However, on D6 acupuncture or progesterone may play a major role in regulating the function of uDCs, such as the expression of mature DCs.

The involvement of DCs in fine-tuning decidual angiogenesis was mainly reported by secreting sFlt1 (VEGFR1) and TGF-*β*1. The vascular activity of VEGF is known to be controlled by sFlt1. In addition, decidual DCs specifically decrease vascular permeability and promote perivascular maturation of implanted sites by secreting sFlt1 [9]. TGF-*β*1 has a protective effect on endothelial cells. It could ensure that sFlt1 only affects VEGF activity and prevent damage of vascular endothelial cells caused by sFlt1 [9]. DCs are not the only VEGF-secreting cells, and our in vitro data represent the ability of uDCs to secrete VEGF. Our results indicated that after COH the level of VEGF secreted by uDCs increased on D4 but decreased on D8, which was consistent with the endometrial expression seen in vivo. This indicates that, during peri-implantation, the secretory state of uDCs is consistent with the microenvironment of angiogenesis. No significant differences were observed for VEGF levels on D6. In addition, no significant differences were found in sFLt-1 secretion by uDCs among the five groups on D4, D6, and D8. Based on the above findings, we believed that the VEGF and sFlt-1 data could perfectly explain the proliferation, tube formation, and migration results. For example, treatment with acupuncture or progesterone did not interfere with sFLT-1 secretion by uDCs but could regulate the secretion of VEGF by uDCs. This may involve a novel mechanism of acupuncture participating in the regulation of angiogenesis in COH rats.

IL-15 and IL-18 are important cytokines, which are secreted by DCs and play important roles in the regulation of natural killer (NK) cell differentiation and proliferation [34]. Nearly half of human decidua DC-SIGN^+^ DCs are in close contact with CD56^+^ NK cells, and a pregnancy-specific interaction exists between the two cell types through cell-cell contact and cytokine secretion to corporately participate in immune tolerance during pregnancy [35]. Our results showed that, after COH, levels of IL-15 and IL-18 secreted by uDCs decreased on D4, D6, and D8; however, the regulatory effects of acupuncture and progesterone were not identical. Both acupuncture and progesterone could not increase levels of IL-15 on D4, and progesterone could increase IL-15 levels on D6 and D8, while acupuncture could only increase IL-15 on D8. Moreover, progesterone treatment increased IL-18 levels on D4 and D6, while acupuncture could only increase IL-18 levels on D8. The above results provide evidence that acupuncture and progesterone regulate the functions of uDCs. In addition, the expressions of VEGF protein and mRNA as well as the proliferation of group P on D8 were not significantly higher compared to that of group M. This may indicate that the effective mechanism of acupuncture and progesterone is not the same. In view of the lack of other literature support, we were unable to make a definite conclusion. However, based on our current findings we speculate that acupuncture or progesterone regulates the ability of uDCs to secrete IL-15 and IL-18, which further regulate the function of NK cells.

Because the endometrial material obtained was not much and the proportion of uDCs in the lymphocytes of uterus was low, we had to use most of the rat uterus tissue for FCM and MACS experiments. It was also proved by our study that there was only tens of thousands of uDCs after MACS, which made it difficult to further verify the results of ELISA by PCR and Western blot. When coculturing, a small number of HUVECs in the transwell could pass through the chamber and fall on the bottom of the lower chamber, where an unknown interaction may occur, which is challenging for performing further research on uDCs after migration assay. However, we found that uDCs with high purity can be obtained by labeling FITC-labeled OX-62 antibodies with magnetic beads, which may provide a methodological reference for further studies. In conclusion, future studies should focus on the effects of acupuncture on the function of immune cells including DCs and NKs during peri-implantation to provide additional scientific explanation for the application of acupuncture in IVF-ET.

## Figures and Tables

**Table 1:** Comparison of mating rate, pregnancy rate, and embryo number.

Group	Mating rate of all rats	Pregnancy rate (D6 and D8)	Embryo number (D8)
N	98.33% (59/60)	100% (47/47)	13.01 ± 1.88 (*n* = 24)
M	95.00% (57/60)	42.56% (20/47)^*∗∗*^	24.30 ± 5.46 (*n* = 10)^*∗∗*^
P	90.00% (54/60)	65.12% (28/43)^#^	16.14 ± 6.10 (*n* = 14)^#^
A	95.00% (57/60)	69.57% (32/46)^##^	15.83 ± 6.01 (*n* = 17)^#^
A + P	91.67% (55/60)	69.77% (30/43)^##^	17.17 ± 3.89 (*n* = 15)^#^

Value = mean ± SD.

*∗∗* represents that there is significant difference between M and N groups (*P* < 0.01).

# or ## represents that there is significant difference when P, A, or A + P is compared with M group (*P* < 0.05 or *P* < 0.01).

**Table 2:** Comparison of expression rate of uDCs by flow cytometry.

Group	D4 (%)	D6 (%)	D8 (%)
N	2.62 ± 0.69 (*n* = 5)	2.17 ± 0.35 (*n* = 5)	2.98 ± 0.69 (*n* = 5)
M	8.88 ± 0.72 (*n* = 5)^*∗∗*^	6.59 ± 0.71 (*n* = 5)^*∗∗*^	1.19 ± 0.39 (*n* = 5)^*∗∗*^
P	3.20 ± 1.84 (*n* = 5)^##^	5.37 ± 1.17 (*n* = 5)^#^	2.73 ± 0.50 (*n* = 5)^##^
A	5.35 ± 2.54 (*n* = 5)^##^	3.73 ± 0.76 (*n* = 5)^##^	2.97 ± 0.84 (*n* = 5)^##^
A + P	4.81 ± 0.78 (*n* = 5)^##^	4.11 ± 1.10 (*n* = 5)^##^	3.38 ± 0.83 (*n* = 5)^##^

Value = mean ± SD.

*∗∗* represents that there is significant difference between M and N groups (*P* < 0.01).

# or ## represents that there is significant difference when P, A, or A + P is compared with M group (*P* < 0.05 or *P* < 0.01).

**Table 3:** Comparison of the number of lymphocytes before MACS.

Group	D4 (×10^5^ cells)	D6 (×10^5^ cells)	D8 (×10^5^ cells)
N	4.3 ± 0.24 (*n* = 5)	5.5 ± 0.71 (*n* = 5)	9.5 ± 0.53 (*n* = 5)
M	4.3 ± 0.40 (*n* = 5)	5.5 ± 0.44 (*n* = 5)	9.8 ± 0.72 (*n* = 5)
P	4.1 ± 0.34 (*n* = 5)	5.8 ± 0.59 (*n* = 5)	9.7 ± 0.35 (*n* = 5)
A	4.2 ± 0.32 (*n* = 5)	5.5 ± 0.59 (*n* = 5)	9.7 ± 0.38 (*n* = 5)
A + P	4.0 ± 0.32 (*n* = 5)	5.9 ± 0.59 (*n* = 5)	9.9 ± 0.53 (*n* = 5)

Value = mean ± SD.

**Table 4:** Comparison of the number of HUVECs after endothelial migration test by uDCs in vitro.

Group	D4	D6	D8
N	476.1 ± 88.8 (*n* = 5)	498.1 ± 163.0 (*n* = 5)	467.0 ± 16.7 (*n* = 5)
M	1032.4 ± 218.7 (*n* = 5)^*∗∗*^	452.2 ± 63.2 (*n* = 5)	165.1 ± 73.9 (*n* = 5)^*∗∗*^
P	574.8 ± 82.6 (*n* = 5)^##^	482.5 ± 132.1 (*n* = 5)	517.7 ± 27.4 (*n* = 5)^##^
A	717.2 ± 153.5 (*n* = 5)^##^	467.2 ± 139.7 (*n* = 5)	527.6 ± 166.1 (*n* = 5)^#^
A + P	677.2 ± 239.4 (*n* = 5)^##^	456.9 ± 82.3 (*n* = 5)	572.3 ± 71.4 (*n* = 5)^##^

Value = mean ± SD. The value means the average number of HUVECs per 100x field of view.

*∗∗* represents that there is significant difference between M and N groups (*P* < 0.01).

# or ## represents that there is significant difference when P, A, or A + P is compared with M group (*P* < 0.05 or *P* < 0.01).

**Table 5:** Comparison of total tubes and total tube length after umbilical vein endothelial tube formation test by uDCs culture supernatant.

	Group	D4	D6	D8
Total tubes	N	33.3 ± 4.7 (*n* = 3)	50.3 ± 4.9 (*n* = 3)	78.0 ± 5.3 (*n* = 3)
	M	66.7 ± 8.7 (*n* = 3)^*∗∗*^	43.3 ± 8.3 (*n* = 3)	27.7 ± 2.1 (*n* = 3)^*∗∗*^
	P	54.3 ± 4.9 (*n* = 3)^#^	39.3 ± 9.3 (*n* = 3)	52.7 ± 4.5 (*n* = 3)^##^
	A	45.3 ± 10.1 (*n* = 3)^##^	45.7 ± 6.0 (*n* = 3)	52.7 ± 8.1 (*n* = 3)^##^
	A + P	46.0 ± 3.0 (*n* = 3)^##^	35.3 ± 1.5 (*n* = 3)	47.3 ± 4.5 (*n* = 3)^##^
Total tube length (px)	N	14196 ± 2015 (*n* = 3)	18459 ± 1700 (*n* = 3)	25485 ± 1321 (*n* = 3)
	M	27957 ± 3261 (*n* = 3)^*∗∗*^	15996 ± 2493 (*n* = 3)	9608 ± 531 (*n* = 3)^*∗∗*^
	P	22931 ± 1467 (*n* = 3)^#^	14675 ± 2585 (*n* = 3)	17555 ± 1679 (*n* = 3)^##^
	A	19414 ± 4097 (*n* = 3)^##^	17209 ± 2134 (*n* = 3)	17210 ± 2379 (*n* = 3)^##^
	A + P	19607 ± 1179 (*n* = 3)^##^	13344 ± 538 (*n* = 3)	15056 ± 1488 (*n* = 3)^##^

Value = mean ± SD.

*∗∗* represents that there is significant difference between M and N groups (*P* < 0.01).

# or ## represents that there is significant difference when P, A, or A + P is compared with M group (*P* < 0.05 or *P* < 0.01).

**Table 6:** Comparison of the levels of VEGF, sFlt-1, IL-15, and IL-18 of uDCs secreting in vitro.

Cytokines	Group	D4	D6	D8
VEGF (pg/ml)	N	45.1 ± 1.6 (*n* = 5)	46.0 ± 5.6 (*n* = 5)	44.4 ± 5.2 (*n* = 5)
	M	55.6 ± 5.4 (*n* = 5)^*∗∗*^	46.1 ± 6.9 (*n* = 5)	33.9 ± 4.5 (*n* = 5)^*∗∗*^
	P	45.9 ± 6.5 (*n* = 5)^##^	45.0 ± 4.6 (*n* = 5)	41.9 ± 4.0 (*n* = 5)^##^
	A	42.0 ± 2.8 (*n* = 5)^##^	43.9 ± 4.1 (*n* = 5)	48.3 ± 5.4 (*n* = 5)^##^
	A + P	41.5 ± 2.8 (*n* = 5)^##^	44.0 ± 2.5 (*n* = 5)	45.6 ± 6.5 (*n* = 5)^##^
sFlt-1 (ng/ml)	N	0.541 ± 0.049 (*n* = 5)	0.560 ± 0.038 (*n* = 5)	0.574 ± 0.038 (*n* = 5)
	M	0.560 ± 0.034 (*n* = 5)	0.592 ± 0.049 (*n* = 5)	0.620 ± 0.095 (*n* = 5)
	P	0.569 ± 0.039 (*n* = 5)	0.596 ± 0.077 (*n* = 5)	0.592 ± 0.043 (*n* = 5)
	A	0.559 ± 0.030 (*n* = 5)	0.559 ± 0.030 (*n* = 5)	0.606 ± 0.045 (*n* = 5)
	A + P	0.601 ± 0.062 (*n* = 5)	0.620 ± 0.060 (*n* = 5)	0.564 ± 0.063 (*n* = 5)
IL-15 (pg/ml)	N	129.0 ± 18.9 (*n* = 5)	30.0 ± 0.96 (*n* = 5)	24.3 ± 0.6 (*n* = 5)
	M	66.9 ± 9.3 (*n* = 5)^*∗∗*^	25.4 ± 0.8 (*n* = 5)^*∗∗*^	22.1 ± 0.4 (*n* = 5)^*∗∗*^
	P	76.0 ± 12.2 (*n* = 5)	28.2 ± 2.0 (*n* = 5)^##^	23.7 ± 1.1 (*n* = 5)^##^
	A	68.0 ± 6.1 (*n* = 5)	26.8 ± 1.0 (*n* = 5)	23.3 ± 0.8 (*n* = 5)^#^
	A + P	68.6 ± 3.4 (*n* = 5)	27.2 ± 1.6 (*n* = 5)^#^	24.8 ± 0.6 (*n* = 5)^##^
IL-18 (pg/ml)	N	34.7 ± 7.5 (*n* = 5)	39.8 ± 2.6 (*n* = 5)	31.0 ± 4.1 (*n* = 5)
	M	23.0 ± 6.6 (*n* = 5)^*∗*^	31.0 ± 1.3 (*n* = 5)^*∗∗*^	21.7 ± 3.6 (*n* = 5)^*∗∗*^
	P	35.3 ± 10.2 (*n* = 5)^#^	35.5 ± 2.9 (*n* = 5)^#^	26.6 ± 6.4 (*n* = 5)
	A	28.4 ± 6.6 (*n* = 5)	32.3 ± 2.6 (*n* = 5)	29.2 ± 4.2 (*n* = 5)^#^
	A + P	35.0 ± 9.5 (*n* = 5)^#^	35.6 ± 2.7 (*n* = 5)^##^	37.6 ± 6.1 (*n* = 5)^##^

Value = mean ± SD.

*∗* and *∗∗* represents that there is significant difference between M and N groups (*P* < 0.01 or *P* < 0.05).

# or ## represents that there is significant difference when P, A, or A + P is compared with M group (*P* < 0.05 or *P* < 0.01).
